# Supplementary material for: Mothers as facilitators for a parent group intervention for children with Congenital Zika Syndrome: Qualitative findings from a feasibility study in Brazil
Source: PLoS One. 2020 Sep 10;15(9):e0238850. doi: 10.1371/journal.pone.0238850 (PMC7482954; doi:10.1371/journal.pone.0238850)
Supplement: S1 Table — (DOCX) [file pone.0238850.s001.docx]

**Topic guide: facilitator interviews**

Materials & preparation:

- Preparation: Print out this interview guide

**Overall aims:**

To explore how the programme ran

To explore how the programme may integrate into existing services

**Present the information sheet and consent form.**

**Introductions and outlining purpose of today’s interview**

# Reflection on the programme

Overall, how do you feel the programme has gone? What areas did you feel worked the best? What areas do you feel worked the least?

Why do you feel these areas worked well/not so well

**Participant factors** e.g, content not relevant to needs of group

**Facilitator factors** e.g. did not feel confident in a particular subject.

**Organisation factors e.g**, venue location, facilities

**Other?**

How did you feel that the training course prepared you for being a facilitator?

What would you do differently if you were to run the programme again?

How did you feel about the other facilitator? Did the dynamic of having one parent/one health worker work? What was good? What was not good?

# Content of the programme

Which were the best sessions of the programme? What made them good?

Are there subjects that you feel were missing in the training programme? Were there areas that you feel were not needed in the training programme? Are there sessions that need to be improved?

# Impact of the programme

How do you feel the caregivers responded to the programme? Did you notice any changes in any of the participants? What were they?

How did you find the group dynamic? Good things? Prompts: Meeting other mums, being able to share your problems?

Do you think that the group will stay in touch now that the programme has finished?

Did you manage to come to each session? If you missed a session, why was that? Did you find it difficult to carry on after missing a session?

**Joint group activities:**  Is your group planning to organise something after this week? How has that come about? How do you feel about that? How will you keep in touch?

# The role of the home visit

Reflect back on the home visit

What are your views on the home visits – were they easy to arrange? Did you find it useful or not?

# Need of the programme

Do you feel that the programme would be useful for other families in similar situations? Have you met other parents who might make use of the programme? Which type of children should be the target of the intervention?

# Integration and expansion

DO you think there is a need for this programme to expand across other parts of Brazil? If so who do you think should be the organisers of such a programme? **Prompts** ask about health clinics, hospitals or parent groups, community centres

# Other

Thank you for your time and for sharing your experience of the training with us. As we think about conducting this training and home visits with other parents, is there anything else important which I haven’t asked you which you want to share with us? Other recommendations?
