# Supplementary material for: Mothers as facilitators for a parent group intervention for children with Congenital Zika Syndrome: Qualitative findings from a feasibility study in Brazil
Source: PLoS One. 2020 Sep 10;15(9):e0238850. doi: 10.1371/journal.pone.0238850 (PMC7482954; doi:10.1371/journal.pone.0238850)
Supplement: S2 Table — (DOCX) [file pone.0238850.s002.docx]

**Topic guide: Post intervention participant interviews**

Materials & preparation:

- Preparation: print our interviews and summarise key issues to further explore for each individual family based on the last visit.
- Copy of the ‘ladder’

**Overall aims:**

To explore if here have been any changes over the course of the programme

To find out what the families felt about the programme

**Present the information sheet and consent form.**

**Introductions and outlining purpose of today’s interview**

# General health and well-being of the child

How has (insert child’s name) been since the programme started?

Has anything changed? Explore in concrete terms e.g. Functional changes, what brought that about, change in general health of the child, child feeling happier, child able to go to school

For each change – explore further what the caregiver thinks has brought about that change

**Individual factors** e.g, change in knowledge/understanding, a change in practice- what was it?

**Assistive devices e.g**, new chair, different equipment for feeding etc- can I see – how has this helped

**Family level changes**: more support in the family, change in attitudes of family- more help in the houe?

**Wider changes:** **Referrals** and links to other services e.g. access to nutritional support/schools. What did that support look like – can you take me through the last time e.g. last visit from the community nutritionist.

**Other?**

**Livelihoods –** last time I saw you explained that you were not able to pay for X ……….you were not able to work- has this changed? In what way?

How do you feel about these changes?

Insert specific follow up probes for each family which will vary:

Use of the assistive device- can I see the assistive device-what has it allowed the child to do?

Support around nutrition- what did that mean – can they take me through the last visit to the nutritionist, changes in food, feeding practice

**Most Significant Change.** Looking over the changes, which do you think is the *Most Significant Change* for your child? Why? **For You what has been the most important change**

# Changes for you as the caregiver

We have been talking a lot about X, now I want to ask you how you are?

Has life changed for you over the course of the programme? If so, in what way?

Prompts around changes Individual: changes in how you understand things, Family: changes in support, wide support group or community support)

Prompts – now able to work more/less, do household chores, less exhausted? emotionally – understand more, more worried/less worried? Communication – with other families members, get more help from other family members)

- To get a more concrete example – ask about something that has happened to the child recently – such as taking to a family event – how did they feel about that? When the child started to feed better/attend school?

**Re-visit the Ladder.**  A year ago I has this ladder and I ask you to tell me where you thought you were on the ladder – can you remember that (explain the ladder exercise again).

Where do you think you are on the ladder at the moment? Why? Is that an improvement from last year or not? What do you think has helped improve things? What has stopped any improvement? Why do you think life has changed/not changed?

And where would you now put your child on the ladder in terms of the quality of their lives? What would improve it further, and why? What could make it worse, and why?

**Support network?** Who do you go to support? So if you have a problem now with child X (for example refer back to an example from the ladder), who would you go and discuss this with?

In your role as caregiver, what is the **Most Significant Change** for you over the last year?

# The role of the support group

How did you find the group? Good things? Any sessions you have particularly liked? Didn’t like?

Can you tell if there is anything that you value about the group? Probe around not just learning new things and general comments but on the value of being a member with other caregivers. Try to ask if the found the group appropriate/acceptable in terms of venue, mix of participants, content of sessions etc.

- Prompts: Meeting other mums, Being able to share your problems?
- Do you find other mums share their experiences? In what way? Is it just focussing on their child?
- Learning from others?
- Language?
- How easy/difficult did you find putting what you learned in the sessions into action at home?

Did you manage to come to each session? If you missed a session, why was that? Did you find it difficult to carry on after missing a session?

**Joint group activities:**  Is your group planning to organise something after this week? How has that come about? How do you feel about that? How will you keep in touch?

If you were to say ONE thing that was of most importance to you from the support group, what would it be?

Was the family able to attend regularly? And if not, why not (will have the attendance data so probe on families who missed sessions

Now that the group has stopped are you in touch with anyone? Plans to stay in touch? If not, why not?

# The role of the home visit

Reflect back on the time we came to visit you at home.

What are your views on the home visits – how have they worked? Do they help? In what way? Are other family members involved at all, and if so, in what way? Has that made any difference in terms of caring for child X?

# Wider learning in the family/community

(Exploring here how is the learning in the parent groups being translated into changes at home and engagement with other family members)

- Do you feel there is any change in how other family members view your child’s condition? Why? Why not? What does that mean in practice? Who looks after the child when you need to work? How do they know this – perhaps a concrete example
- Refer to the parent handout – has it been useful to have this? Shared with other family members (despite being in English?) have you been able to share any learning from the group?
- Greater understanding in the community? In what way? (huge levels of stigma, beliefs that child a spirit child- what can you say to people? Have you shared any of this information with other neighbours etc.) How did this come about? Do you think it’s really making any difference? (group activities, role of the facilitator)

# Need of the programme

Do you feel that the programme would be useful for other families in similar situations to you? Have you met other parents who might make use of the programme?

# Other

Thank you for your time and for sharing your experience of the training with us. As we think about conducting this training and home visits with other parents, is there anything else important which I haven’t asked you which you want to share with us? Other recommendations?
