# Supplementary material for: Mothers as facilitators for a parent group intervention for children with Congenital Zika Syndrome: Qualitative findings from a feasibility study in Brazil
Source: PLoS One. 2020 Sep 10;15(9):e0238850. doi: 10.1371/journal.pone.0238850 (PMC7482954; doi:10.1371/journal.pone.0238850)
Supplement: Attachment — Submitted filename: Zika article Plos One.pdf [file pone.0238850.s003.pdf]

PLOS ONE

Mothers as facilitators for a parent group intervention for children with congenital zika syndrome: qualitative findings from a feasibility study in Brazil

--Manuscript Draft--

**Manuscript Number:**	PONE-D-20-02401
**Article Type:**	Research Article
**Full Title:**	Mothers as facilitators for a parent group intervention for children with congenital zika syndrome: qualitative findings from a feasibility study in Brazil
**Short Title:**	The feasibility of mothers as facilitators for a group intervention for children affected by zika in Brazil
**Corresponding Author:**	Tracey Smythe<br>London School of Hygiene and Tropical Medicine<br>London, UNITED KINGDOM
**Keywords:**	zika; disability; impairment; parents; mothers; support; caregivers; peer group; community; congenital zika syndrome; qualitative
**Abstract:**	Background<br>The Zika virus outbreak in Brazil (2015-2016) affected thousands of children who were born with congenital zika syndrome (CZS). Families play an important role in their care of children with complex needs, and evidence is lacking on how to best support these families.<br>Objective<br>This study explores the use of mothers as facilitators for a community-based intervention for children with CZS and their caregivers in Brazil.<br>Methods<br>Four facilitators were trained to deliver the intervention, two were mothers of a child with CZS (“expert mothers”) and two were therapists (speech therapist and physiotherapist). The intervention was delivered to three groups. Two psychologists observed the groups and held focus group discussions at the end of each session. They undertook semi-structured interviews post intervention with a purposive sample of caregivers, and with the facilitators. Observation notes were collated and summarised. Transcripts were transcribed and thematically analysed using five elements to assess feasibility: acceptability, demand, implementation, practicality and adaption.<br>Results<br>The use of expert mothers as facilitators was acceptable. Their experiential knowledge was viewed as important for sharing and learning, and supporting and encouraging the group. The intervention was delivered with fidelity by the expert mothers. Equal payment with the therapist enabled the expert mothers to better facilitate the groups, through increased confidence in the value of their role. One expert mother was unable to attend two sessions due to her child being ill. It was important for expert mothers to be local to the community in which the intervention was run, whilst it was acceptable for facilitator therapists to travel further and be involved in several groups.<br>Conclusion<br>The use of expert mothers as facilitators of caregiver groups provides a unique approach to harness the capacity of families to provide care, and is likely to be feasible in similar contexts.
**Order of Authors:**	Tracey Smythe
	Monica Matos
	Julia Reis
	Antony Duttine
	Silvia Ferrite
	Hannah Kuper
**Additional Information:**	

Powered by Editorial Manager® and ProduXion Manager® from Aries Systems Corporation

Question	Response
**Financial Disclosure**<br><br>Enter a financial disclosure statement that describes the sources of funding for the work included in this submission. Review the submission guidelines for detailed requirements. View published research articles from *PLOS ONE* for specific examples.<br><br>This statement is required for submission and **will appear in the published article** if the submission is accepted. Please make sure it is accurate.<br><br>**Unfunded studies**<br>Enter: *The author(s) received no specific funding for this work.*<br><br>**Funded studies**<br>Enter a statement with the following details:<br>• Initials of the authors who received each award<br>• Grant numbers awarded to each author<br>• The full name of each funder<br>• URL of each funder website<br>• Did the sponsors or funders play any role in the study design, data collection and analysis, decision to publish, or preparation of the manuscript?<br>• **NO** - Include this sentence at the end of your statement: *The funders had no role in study design, data collection and analysis, decision to publish, or preparation of the manuscript.*<br>• **YES** - Specify the role(s) played.<br><br>* typeset	The development and testing of the intervention for children with Congenital Zika Syndrome were supported by a grant from Wellcome Trust and the Department for International Development (https://wellcome.ac.uk/) to HK: grant code 206719/Z/17/Z. The funders had no role in the design of the study; in the collection, analyses, or interpretation of data; in the writing of the manuscript, or in the decision to publish the results.
**Competing Interests**<br><br>Use the instructions below to enter a competing interest statement for this submission. On behalf of all authors, disclose any competing interests that could be perceived to bias this work—acknowledging all financial support and any other relevant financial or non-financial competing interests.<br><br>This statement **will appear in the published article** if the submission is	I have read the journal's policy and the authors of this manuscript have the following competing interests:One of the authors (AD) joined the Pan American Health Organisation (PAHO) during the research period. Work on the research study was undertaken outside and separate to his PAHO duties.

accepted. Please make sure it is accurate. View published research articles from *PLOS ONE* for specific examples.<br><br>**NO authors have competing interests**<br><br>Enter: *The authors have declared that no competing interests exist.*<br><br>**Authors with competing interests**<br><br>Enter competing interest details beginning with this statement:<br><br>*I have read the journal's policy and the authors of this manuscript have the following competing interests: [insert competing interests here]*<br><br>* typeset	
**Ethics Statement**<br><br>Enter an ethics statement for this submission. This statement is required if the study involved:<br><br>• Human participants<br>• Human specimens or tissue<br>• Vertebrate animals or cephalopods<br>• Vertebrate embryos or tissues<br>• Field research<br><br>Write "N/A" if the submission does not require an ethics statement.<br><br>General guidance is provided below. Consult the submission guidelines for detailed instructions. **Make sure that all information entered here is included in the Methods section of the manuscript.**	Ethical approval was gained from Instituto de Saúde Coletiva - ISC/UFBA Ethics Ref 2.369.348, Instituto Fernandes Figueira - IFF/ FIOCRUZ - RJ/MS Ethics Ref 2.183.547 and LSHTM Ethics Ref 13608. Written informed consent was acquired from all participants.

Powered by Editorial Manager® and ProduXion Manager® from Aries Systems Corporation

**Format for specific study types**<br><br>**Human Subject Research (involving human participants and/or tissue)**<br>• Give the name of the institutional review board or ethics committee that approved the study<br>• Include the approval number and/or a statement indicating approval of this research<br>• Indicate the form of consent obtained (written/oral) or the reason that consent was not obtained (e.g. the data were analyzed anonymously)<br><br>**Animal Research (involving vertebrate animals, embryos or tissues)**<br>• Provide the name of the Institutional Animal Care and Use Committee (IACUC) or other relevant ethics board that reviewed the study protocol, and indicate whether they approved this research or granted a formal waiver of ethical approval<br>• Include an approval number if one was obtained<br>• If the study involved *non-human primates*, add *additional details* about animal welfare and steps taken to ameliorate suffering<br>• If anesthesia, euthanasia, or any kind of animal sacrifice is part of the study, include briefly which substances and/or methods were applied<br><br>**Field Research**<br><br>Include the following details if this study involves the collection of plant, animal, or other materials from a natural setting:<br>• Field permit number<br>• Name of the institution or relevant body that granted permission	
**Data Availability**<br><br>Authors are required to make all data underlying the findings described fully available, without restriction, and from the time of publication. PLOS allows rare exceptions to address legal and ethical concerns. See the PLOS Data Policy and FAQ for detailed information.	No - some restrictions will apply

Powered by Editorial Manager® and ProduXion Manager® from Aries Systems Corporation

A Data Availability Statement describing where the data can be found is required at submission. Your answers to this question constitute the Data Availability Statement and **will be published in the article**, if accepted.<br><br>**Important:** Stating 'data available on request from the author' is not sufficient. If your data are only available upon request, select 'No' for the first question and explain your exceptional situation in the text box.<br><br>Do the authors confirm that all data underlying the findings described in their manuscript are fully available without restriction?	
**Describe where the data may be found in full sentences. If you are copying our sample text, replace any instances of XXX with the appropriate details.**<br><br>• If the data are **held or will be held in a public repository**, include URLs, accession numbers or DOIs. If this information will only be available after acceptance, indicate this by ticking the box below. For example: *All XXX files are available from the XXX database (accession number(s) XXX, XXX.).*<br>• If the data are all contained **within the manuscript and/or Supporting Information files**, enter the following: *All relevant data are within the manuscript and its Supporting Information files.*<br>• If neither of these applies but you are able to provide **details of access elsewhere**, with or without limitations, please do so. For example:<br><br>*Data cannot be shared publicly because of [XXX]. Data are available from the XXX Institutional Data Access / Ethics Committee (contact via XXX) for researchers who meet the criteria for access to confidential data.*<br><br>*The data underlying the results presented in the study are available from (include the name of the third party*	We as authors are fully supportive of sharing our study data. Data from this study, which consist of confidential interviews with mothers of children with zika syndrome, may be accessed by request only. Requests for data access will be considered and responded to within one month of the request and de-identified datasets will be made available electronically. Requests for data may be sent to: disabilitycentre@lshtm.ac.uk.

and contact information or URL).<br>• This text is appropriate if the data are owned by a third party and authors do not have permission to share the data.<br><br>* typeset	
Additional data availability information:	Tick here if your circumstances are not covered by the questions above and you need the journal's help to make your data available.

Powered by Editorial Manager® and ProduXion Manager® from Aries Systems Corporation

Manuscript

Click here to download Manuscript mothers as facilitators_manuscript_27Jan.docx

Title page

Full title of manuscript: Mothers as facilitators for a parent group intervention for children with congenital zika syndrome: qualitative findings from a feasibility study in Brazil

Short title: The feasibility of mothers as facilitators for a parent group intervention for children affected by zika in Brazil

Authors: Tracey Smythe[1], International Centre for Evidence in Disability, London School of Hygiene & Tropical Medicine, tracey.smythe@lshtm.ac.uk

Monica Matos, Masters candidate at Collective Health's Institute, Universidade Federal da Bahia, Salvador, Brazil, monicamatos@gmail.com

Julia Reis, Department of Child Psychology, Rehabilitation Institute of Bahia, Salvador, Brazil, juliareisesilva@gmail.com

Antony Duttine, International Centre for Evidence in Disability, London School of Hygiene & Tropical Medicine, antony.duttine@lshtm.ac.uk

Silvia Ferrite, Department of Speech and Hearing Sciences, Federal University of Bahia, Salvador, Brazil, ferrite@ufba.br

Hannah Kuper, International Centre for Evidence in Disability, London School of Hygiene & Tropical Medicine, Hannah.kuper@lshtm.ac.uk

[1]Corresponding Author: International Centre for Evidence in Disability, London School of Hygiene & Tropical Medicine, Keppel St, Bloomsbury, London WC1E 7HT

Abstract: 299 words

Key words: disability, impairment, parents, mothers, support, caregivers, peer group

Abstract

Background

The Zika virus outbreak in Brazil (2015-2016) affected thousands of children who were born with congenital zika syndrome (CZS). Families play an important role in their care of children with complex needs, and evidence is lacking on how to best support these families.

Objective

This study explores the use of mothers as facilitators for a community-based intervention for children with CZS and their caregivers in Brazil.

Methods

Four facilitators were trained to deliver the intervention, two were mothers of a child with CZS ("expert mothers") and two were therapists (speech therapist and physiotherapist). The intervention was delivered to three groups. Two psychologists observed the groups and held focus group discussions at the end of each session. They undertook semi-structured interviews post intervention with a purposive sample of caregivers, and with the facilitators. Observation notes were collated and summarised. Transcripts were transcribed and thematically analysed using five elements to assess feasibility: acceptability, demand, implementation, practicality and adaption.

Results

The use of expert mothers as facilitators was acceptable. Their experiential knowledge was viewed as important for sharing and learning, and supporting and encouraging the group. The intervention was delivered with fidelity by the expert mothers. Equal payment with the therapist enabled the expert mothers to better facilitate the groups, through increased confidence in the value of their role. One expert mother was unable to attend two sessions

due to her child being ill. It was important for expert mothers to be local to the community in which the intervention was run, whilst it was acceptable for facilitator therapists to travel further and be involved in several groups.

Conclusion

The use of expert mothers as facilitators of caregiver groups provides a unique approach to harness the capacity of families to provide care, and is likely to be feasible in similar contexts.

Introduction

Developmental disabilities affect at least 50 million children under the age of 5 years and are a major contributor to child and adult morbidity in low and middle income countries [1]. Children with developmental disabilities have multiple impairments (e.g. cognitive, physical, visual) that have a long-term influence on health and development [2]. Families play an important role in care for these children, and caregivers of children with disabilities often experience high levels of stress, anxiety, depression, physical exhaustion, and discrimination [3-8]. These experiences contribute to decreased quality of life compared to caregivers of non-disabled children, and may result in reduced effectiveness of parenting [9, 10]. Evidence is lacking on how best to support these families, particularly those living in resource limited settings where healthcare providers with the appropriate expertise may be lacking. Support programmes in partnership with health professionals are increasingly being used to try to fill these gaps, but they often have a top-down approach, rather than drawing on the expertise of family members.

The World Health Organisation (WHO) recommends a range of interventions for children with or at risk of developmental disabilities, such as empowerment of caregivers and a shift from child-centred to family-centred care, to provide optimal stimulation for development in a safe, stable and nurturing environment [11]. Family-centred care focusses on an approach to services in which families are recognised as the experts on their child, and work with service providers to make informed decisions about their child's care. There is a growing body of evidence on how best to offer family-centred care in lower resourced settings, and on the benefits of participatory peer learning [12]. Peer support interventions are hypothesised to work by increasing the amount of social support available to parents

and carers of children with complex needs, and providing that support in a form which is most useful and acceptable to participants [13]. As an example, women's groups that practice participatory learning and action have been shown to improve maternal and child health and empower women (14), and are recommended by the WHO to reduce newborn mortality and improve health in low resource settings (16). There are a number of potential advantages to support groups for caregivers of children with developmental disabilities. Participatory peer learning in groups provides caregivers opportunities to practice activities with their child and receive feedback, and has greater benefits compared with providing parenting information only (11). Support groups provide positive benefits to child development and wellbeing, and family functioning [14], improve caregiver understanding, confidence and self-esteem, and reduce self-blame [15]. Additionally, support groups may offer an important social safety net for caregivers who are excluded in their communities [15].

One context in which peer support group interventions could be relevant but have not yet been explored is for the care of children with congenital zika syndrome (CZS). In January 2016, the WHO reported more than three thousand cases of microcephaly suspected of being related to Zika Virus (ZIKV) in Brazil [16]. The causal link between ZIKV infection and microcephaly has since been established [17, 18]. Congenital infection with ZIKV is linked to other abnormalities besides microcephaly, including neurological conditions, ophthalmic abnormalities, hearing loss and bone and joint disorders [19-21], now collectively called CZS. Children with CZS are likely to have complex intellectual, physical and sensory impairments over their lifetime [22]. Additionally, a wider spectrum of developmental impairments may yet manifest in children who were exposed to Zika in utero as they continue their development [23]. Provision of family and supportive services is essential to meet the

broader needs of these children and caregivers, and to complement clinical and other services available in Brazil.

One potentially effective strategy to provide these services is through caregiver support programmes. We used this approach to develop a peer group support intervention ("Juntos", meaning 'together' in Portuguese) to provide psychosocial support and improve the skills of caregivers of children with CZS in Brazil, to optimally care for their child [24, 25]. The Juntos programme consists of ten sessions offered over a period of 3 months held in the local community. Each session includes ice-breaker activities, practical sessions and group discussions, and a psychological support component, and lasts approximately 4 hours. The content of the programme takes learning from health concerns that affect children with cerebral palsy (CP) [26], which is a similar developmental disability to CZS. The programme includes participatory learning about care practices, such as feeding, positioning, communication, everyday activities, play and early stimulation, in addition to disability rights and inclusion (Table 1). Every session includes an emotional support activity to provide a safe environment in which to stimulate open and supportive discussion between caregivers about their successes and difficulties. Programme material is available from www.ubuntu-hub.org

Table 1. Juntos intervention module topics.

Module number and title	Topics covered
1: Introduction	About the programme<br>Information about Zika and Congenital Zika syndrome<br>How to find information<br>Personal stories
2: Our child	Introducing your close family and friends<br>Development milestones for young children

	Determining your child’s progress<br>Managing irritability and crying
3: Positioning and moving	How to position children who need assistance<br>How to assist children to learn to move
4: Eating and drinking	Feeding challenges<br>Practical skills to address challenges for your child
5: Communication	Importance of communication<br>Practical advice to help your child communicate
6: Play and early stimulation	Importance of play for children to develop and learn<br>Early stimulation<br>Making simple toys<br>Inclusion of play in the family and broader community
7. Everyday activities	How to use everyday activities to help your child develop<br>Managing seizures
8. Uniting our voices	Understand the context of disability rights<br>Education<br>Communicating with your health team<br>Advocating<br>Thoughts and feelings
9. Our community	Who is in your community<br>Common barriers to inclusion<br>Addressing negative attitudes and exclusion<br>Thoughts and feelings<br>Social Activity
10. Next steps	Summing up<br>Planning next steps for yourself and the group

The existing caregiver support programmes for children with CP relied on therapists as facilitators, but within Juntos we pilot-tested the use of a parent of child with CZS (“expert mother”) working alongside a therapist facilitator. This approach was used to encourage a more participatory process and atmosphere of sharing between the caregivers. There were concerns about the acceptability and practicality of this approach and we therefore aimed to explore the feasibility of the use of mothers as facilitators for the community based group intervention for children with CZS and their caregivers in Greater Salvador, Bahia.

Materials and methods

Ethical approval was gained from Instituto de Saúde Coletiva - ISC/UFBA Ethics Ref 2.369.348, Instituto Fernandes Figueira - IFF/ FIOCRUZ - RJ/MS Ethics Ref 2.183.547 and LSHTM Ethics Ref 13608. Written informed consent was acquired from all participants.

Study design

This study is part of a larger pre post intervention design in Greater Salvador and Rio de Janeiro, which has been described in detail previously [24]. This qualitative study was undertaken between August 2017 and May 2018 in three municipalities of Greater Salvador, Bahia. Data were collected from observation of the caregiver groups (n=26), focus group discussions with caregivers (n=26), post-intervention questionnaires (n=20) and semi-structured interviews (n=13) with group facilitators and purposively selected caregivers.

Participants and setting

Participants were caregivers of children with neurologist-confirmed CZS in Great Salvador, Bahia. For the purposes of this study, we used CZS to describe any child with impairments that can be directly attributed to Zika. The impairments in children included either mild cognitive, communication or functional skill delay and ranged to severe delay in all three developmental categories. The average group size was 8-10 caregivers. Each group met weekly for approximately four hours for ten sessions. The groups were facilitated by one expert mother paired with one therapist (speech therapist or physiotherapist) who had undergone a one-week facilitator training programme; in total two mothers and two therapists were trained. The facilitators were identified and selected by the study site co-ordinator. The hypothesised role of the expert mother was to facilitate a participatory and

equal atmosphere, and encourage the sharing of learning between caregivers. For example, the expert mother led the emotional support activity to help to stimulate open and supportive discussion between caregivers about their successes and difficulties in the previous week. The role of the therapist was to facilitate the technical aspects of skill acquisition and practice of techniques, such as feeding positions. Both facilitators were paid the same amount.

Data collection

The reasons for doing the research were explained and data were collected by two female Brazil based research assistants (psychologists). Neither of the psychologists worked in the clinical area or had prior knowledge of the participants. Three techniques were used for the collection of qualitative data. First, participant observations of all ten sessions for each of the three groups were noted during each session and recorded in an Excel file. An observation checklist was used to assess fidelity of delivery of the intervention by the facilitators. In addition, observation of the ice-breaker activities, practical sessions and group discussions provided an opportunity to examine the interaction of the expert mothers with the participants and facilitator therapist during each session. The psychologists observing the sessions wrote detailed field-notes. Second, the psychologists (one per site) facilitated focus group discussions related to the content and processes of the session (approximately forty-five minutes) at the end of each session. The psychologists took comprehensive notes of the discussion with the participants. Third, semi-structured interviews with both facilitators of the group and a purposively selected sample of participants, to ensure geographic spread and a range of caregiver ages, were undertaken for triangulation of data. The interview guide was piloted for understanding and interviews

lasted on average 1 hour, ranging between 50 minutes and 1 hour 20 minutes. Interviews were designed to explore participants' motivation for attending the intervention, their views around how they were approached to participate in 'Juntos', how they viewed the programme itself, and aspects around engagement were explored. ~~The interviews were recorded through audio techniques~~. Focus groups and interviews were conducted in Portuguese.

Data management and analysis

Observation data and comments that related to the role of facilitators from focus group discussions were collated in an Excel sheet (Microsoft Excel 2000 (Microsoft Inc., Redmond, Washington) after each group intervention by one lead UK based researcher, a specialized paediatric physiotherapist trained in qualitative and quantitative techniques (TS). The two psychologists (MM and JR) transcribed and translated the audio-recorded data from the semi-structured interviews. We used a framework based on a model proposed by Bowen et al (2010) [27], designed to assess the feasibility of public health interventions, to evaluate the participatory group intervention from the first five facets of acceptability, demand, implementation, practicality and adaption. The remaining three areas of limited efficacy, expansion and integration are to be explored in future studies. Words and phrases relating to the effect of having an expert mother facilitate the intervention were identified and coded to the feasibility framework. They were discussed between the two psychologists for agreement and checked and verified by TS. Consensus was gained through discussion. We undertook a narrative synthesis of the findings. Illustrative quotes are presented in the findings. We reported the results according to the consolidated criteria for reporting

qualitative research (COREQ) [28], which is a 32-item checklist for interviews and focus group.

Results

Three Juntos groups were convened in Greater Salvador (in Simões Filho, Lauro de Freitas, Camaçari) between August 2017 and May 2018. A total of 27 families and their children enrolled. Thirty-eight participants (mother, father or other caregiver) attended at least one session. The two psychologists undertook 120 hours of observation. Thirty focus group discussions were convened. Nine semi-structured participant interviews and four semi-structured facilitator interviews were undertaken.

Acceptability

Acceptability considers how the recipients react to the inclusion of expert mothers. Support from expert mothers was considered to be highly valued by the participants in this study. Both participants and facilitators talked about the benefits of including the expert mother and the role of the expert mother was viewed as acceptable in two ways; (1) promoting sharing and learning from each other, and (2) supporting and encouraging each other.

(1) Sharing and learning from each other

The psychologists observed that inclusion of the expert mothers contributed to an environment in which participants shared their own experiences and listened to others in similar situations. Expert mothers' knowledge and expertise from first-hand experiences was shared with others in similar situations, and an example of sharing from the perspective of the expert mother includes:

"*I experienced something really incredible when a mother did not believe that her daughter was capable of doing something and, at that moment, I remembered that my son had had the same difficulty. I knew that her child was capable, so I shared strategies that I learnt for my child and she was able to do it*." (Expert mother 01)

Expert mothers led by example to increase the inclusion of other family members and their communities to assist with care of their child. One expert mother required support from her husband to facilitate a group and she explained to the group:

"*to be here [facilitating the group intervention] I had to train my husband*" (Expert mother 02)

This example demonstrated to other caregivers what may be possible when seeking support from other family and community members.

Caregivers and expert mothers reported ~~finding~~ a shared social identify. This was evident through the shared experience of their children's unpredictable medical needs, which was faced by the expert mothers and the participants. For example, an expert mother was unable to attend one session due to her child being ill:

"*I cannot be here today because I have to be in hospital with my child*" (Expert mother 01)

Fostering a shared sense of identity led caregivers to meet outside of the support programme after the three months concluded, expanding their social ~~and~~ support network.

(2) Supporting and encouraging each other

The expert mothers were regularly observed to encourage parents that "you know better than you believe". Through the process of participatory peer learning, one expert mother told us:

> "*By learning and acting as an expert mother, Juntos enabled me to find out about so many new things and people, who will forever remain part of my story. We had so many questions, and together we mothers were heard and we listened to each other. It was a very important change because normally families believe that they should just listen to professional therapists."* (Expert mother 01)

The expert mothers appreciated how difficult it could be to meet the needs of a child with a disability. Participants reported that acceptance from the expert mothers helped to increase their self-belief and confidence in their own ability to care for their child. This was facilitated through being seen as on an 'equal level':

> "*At the first sessions I felt like a mere spectator. I was only there to "learn" to handle my daughter. But, what does that mean, "learn"? Does it mean that I, as her carer, who is with her every second of the day, has nothing to offer? …we ran the sessions so that the families had equally important roles*." (Expert mother 02)

The benefits of including the expert mother were also articulated by the therapist facilitator, ~~reducing the traditional medical hierarchy~~. One facilitator therapist reported change in how she provided support to families:

> "*Being a facilitator is not easy, you need to be available to care…sharing the facilitator space with a mother enabled me to get closer to a reality that I had only*

> *experienced from a distance. Hearing from other carers and sharing so much knowledge, which only they have, was transforming*." (Facilitator therapist 02)

The expert mothers were viewed as acceptable to participants as they encouraged the provision of mutual support. This provided a strong motivation for participants to offer the same support to other caregivers, which they had benefitted from themselves:

> *"No one has asked before about my hopes or dreams. Today I see if I am well then I can take better care of my child. Others should know this too. We must take care of ourselves…it is important to help us understand this. I now tell others about this often."* (Participant 04)

There was no objection from other mothers about payment of expert mothers as facilitators. Limited acceptability may occur when caregivers are concerned with the comparison between their own and the child of the expert mother. However, this was not experienced within these groups.

Demand

~~In this study~~, demand for the intervention is indicated by the documenting of activities by the expert mother in the caregiver groups. Expert mothers were wanted by the other caregivers, and participants reported valuing the opportunity to share personal experiences and problem solve together with someone who understood their circumstances. The expert mothers provided an example of what may be possible through their lived experience, which indicates the way the ~~group~~ was delivered was appropriate and accessible to carers. This experience was underpinned by the importance of building hope and confidence. One participant acknowledged:

> “*if she is telling me, then I can do it*.” (Participant 07)

Additionally, as the facilitator pairs worked together over time, the value of the expert mothers became more clear to the therapist facilitator. The therapist facilitators voiced wanting to continue facilitating groups of parents alongside an expert parent:

> *“Working together with a mother facilitator, my senses are much more attentive to each family’s story, and these stories have acted on me and changed me, both personally and professionally.”* (Facilitator Therapist 02)

Expert mothers were viewed to improve the understanding of the need of families and the professional abilities of the therapist facilitator to address these needs.

Implementation

Implementation concerns the extent, likelihood, and manner in which facilitation by the expert mother can be fully implemented as planned and proposed, often in an uncontrolled design. The intervention content and processes were delivered with fidelity by the expert mothers; the content (e.g. topics covered from module one to ten, the emotional support activity) and processes (e.g. participatory learning, adult learning techniques) of the intervention were used to a large extent by the expert mothers. The expert mothers enacted their expected role within the group. The psychologists observed that they facilitated a participatory and equal atmosphere, and encouraged the sharing of learning between caregivers. Both expert mothers were purposively selected by the site co-ordinator, ~~with~~ a similar socio-economic level as the group participants, and had completed high school at a minimum as they were required to follow a facilitator manual to deliver the intervention. All participants were literate. The therapist and expert mothers were paid and

this may have influenced their commitment to the programme. The expert mothers filled their planned role (described above) and this differed from the role of the therapist, who facilitated the technical aspects of skill acquisition, such as feeding positions. Both facilitators contributed to practice of techniques. However, changes to the on-going support through increased access to mentors was required. For example, the expert mothers led the emotional support activity as part of every session, but they ~~do~~ not have a mental health ~~background~~. The observing psychologists identified the need to provide regular psychological support to the expert mothers after delivering each session. The expert mothers were therefore provided with weekly mentoring by the psychologists. This input deviated from the limited support that was initially planned. The relationship between the facilitator therapist and the expert mother developed through delivery of the programme and sustained by enthusiastic therapists that advocate for family centred services.

Practicality

Practicality considers the extent to which facilitation by the expert mother can be implemented when resources, time, commitment, or some combination thereof are constrained in some way. There were two occasions when an expert mother was unable to attend. The weekly sessions were held on different week days and the other expert mother was able to assist with one session. In the other session, it was run only by the facilitator therapist. The expert mother in these cases was unable to attend due to her child being ill. Over the three-month period, the majority of caregivers missed at least one session due to illness of their child. Transport costs were reimbursed for the expert mothers as well as the participants. The expert mother was paid the same as the facilitator therapist (2,100BRL/month, approximately USD500), however this cost decision was initially viewed

as unfavourable by the administration team due to precise costing guidelines provided for therapists and relevant experience, with the suggested payment for expert mothers as 1,500BRL/month, approximately USD360. The estimate of salary cost for one therapist to deliver 10 group sessions over three months is 6,300BRL (approximately USD1,500) and the incremental cost of including an expert mother would be 6,300BRL (approximately USD1,500) per programme. It is likely that this equal payment enabled the expert mothers to better facilitate the groups, being more confident of the value of their role in a pair with the therapist.

Adaption

Adaptation focuses on how the role of the expert mother may need to be adapted for a new situation. In order to train the expert mother in delivery of Juntos, a standardised 5-day facilitators training programme with ongoing weekly mentoring guidelines was developed in partnership with the expert mothers and facilitator therapists after the first pilot. The Juntos materials were further developed to include videos of expert mothers and fathers demonstrating techniques such as brushing teeth and play. These videos were reported as helpful to expert mothers to further demonstrate techniques to participants. Future consideration for scale up includes accounting for resource-limited contexts with fewer number of therapists. It is likely that adaption of the training to include a greater number of expert mothers compared to the number of facilitator therapists may be required; it was reported as important for the expert mother to be local to the community in which the community-based group intervention is run, whilst it is acceptable for the facilitator therapist to travel further and be involved in a greater number of groups.

Discussion

The role of mothers as facilitators in community group interventions is likely to be feasible through participatory peer learning to improve care for children with developmental disabilities. The use of expert mothers was acceptable for participants and facilitator therapists. Their role was seen as important to share and learn together, and to provide support and encouragement. Expert mothers are relevant in the context of family-centred services because their role emphasises that families are the experts on their child and their family's situation. The equity in partnership between expert mothers and facilitator therapists was by design and the therapist recognised that each facilitator brought their own unique expertise to the group that the other did not possess.

Our findings contribute to the growing body of evidence of participatory peer learning to improve child care and support caregivers' psychological and emotional wellbeing [29]. Examples in lower resourced settings include the development of caregiver groups in Ghana, which were guided by a theory of change [26]. A sense of belonging and creation of common ground was theorised to provide an environment to improve the knowledge and skills of carers through a social support network. This approach, in turn, was found to improve the quality of life of children with cerebral palsy and their caregivers [26]. This theory of change is key to suggest why using expert mothers may be important. With regards maternal and child health, a systematic review and meta-analysis of randomised controlled trials undertaken in Bangladesh, India, Malawi, and Nepal of women's groups practicing participatory learning and action were found to improve maternal and neonatal survival [30]. The women's groups aimed to increase appropriate care-seeking, and appropriate home prevention and care practices for mothers and newborns [31]. The women's groups drew on principles of Paulo Freire's work [32], namely: (i) health challenges are often rooted in powerlessness, and can be addressed by social empowerment; (ii)

including dialogue and problem solving in health education is more empowering than information giving; and (iii) communities can develop critical consciousness to recognise and address the underlying social and political determinants of health. While there is no single recognised theory of how women's groups practising participatory learning and action achieve their health impacts [33], group participation and membership offers a valuable social support network to navigate medical hierarchies, and may contribute to change in care practices through increasing confidence of caregivers.

The two themes from acceptability - sharing and learning from each other and supporting and encouraging each other - include stories of positive change, however the feasibility of the use of expert mothers may have contextual limitations and require additional modification to implementation. It is difficult to identify how much change reported by the participants depended on specific personal characteristics of individual expert mothers, and how much on the use of an expert mother of any kind. The success of the facilitator partnership is likely dependent on both personality and perspective of the pair. In the selection of expert mothers in Bahia, Brazil, selection characteristics included (i) a similar socioeconomic level to participants, (ii) having a child with a pattern of severity of CZS, (iii) a willingness to help other families, (iv) an active participant in disability rights, and (v) tolerance to personal differences and perspectives (e.g. religion). These characteristics may link to a sense of belonging and creation of common ground. Future research should investigate the experience of the person offering support and the impact of peer support on caregivers' relationships with health care professionals. In addition, the role of equal payment warrants further attention.

This study has limitations. We explored feasibility of use of two expert mothers in only one setting. We did not compare different strategies of delivery. Nor did we explore in detail what aspects of expert mother were critical to success. A subset of participants was selected to participate in semi-structured interviews, however the triangulation of findings between participants, expert mothers and facilitator therapists gives us confidence in our results.

Conclusion

Caregivers with similar life experiences may provide an innovate community support to families in resource limited settings. The use of expert mothers in a participatory group setting offers a unique approach to harness the capacity of families to provide care for their child and may be feasible in similar settings. Future directions include expansion of the intervention to include children with broad developmental disabilities.

Acknowledgments

N/A

Ethics and consent

Ethical approval was gained from Instituto de Saúde Coletiva - ISC/UFBA Ethics Ref 2.369.348, Instituto Fernandes Figueira - IFF/ FIOCRUZ - RJ/MS Ethics Ref 2.183.547 and LSHTM Ethics Ref 13608. Written informed consent was acquired from all participants.

References

1. Global Research on Developmental Disabilities C. Developmental disabilities among children younger than 5 years in 195 countries and territories, 1990-2016: a systematic analysis for the Global Burden of Disease Study 2016. Lancet Glob Health. 2018;6(10):e1100-e21. doi: 10.1016/S2214-109X(18)30309-7. PubMed PMID: 30172774; PubMed Central PMCID: PMCPMC6139259.

2. Black MM, Walker SP, Fernald LCH, Andersen CT, DiGirolamo AM, Lu C, et al. Early childhood development coming of age: science through the life course. Lancet. 2017;389(10064):77-90. Epub 2016/10/09. doi: 10.1016/S0140-6736(16)31389-7. PubMed PMID: 27717614; PubMed Central PMCID: PMCPMC5884058.

3. Bunning K, Gona JK, Newton CR, Hartley S. The perception of disability by community groups: Stories of local understanding, beliefs and challenges in a rural part of Kenya. PLoS One. 2017;12(8):e0182214. doi: 10.1371/journal.pone.0182214. PubMed PMID: 28771637; PubMed Central PMCID: PMCPMC5542666.

4. Gona JK, Mung'ala-Odera V, Newton CR, Hartley S. Caring for children with disabilities in Kilifi, Kenya: what is the carer's experience? Child Care Health Dev. 2011;37(2):175-83. doi: 10.1111/j.1365-2214.2010.01124.x. PubMed PMID: 20645990; PubMed Central PMCID: PMCPMC3072473.

5. Nakamanya S, Siu GE, Lassman R, Seeley J, Tann CJ. Maternal experiences of caring for an infant with neurological impairment after neonatal encephalopathy in Uganda: a qualitative study. Disabil Rehabil. 2015;37(16):1470-6. doi: 10.3109/09638288.2014.972582. PubMed PMID: 25323396; PubMed Central PMCID: PMCPMC4784505.

6. Tilahun D, Hanlon C, Fekadu A, Tekola B, Baheretibeb Y, Hoekstra RA. Stigma, explanatory models and unmet needs of caregivers of children with developmental disorders in a low-income African country: a cross-sectional facility-based survey. BMC Health Serv Res. 2016;16:152. doi: 10.1186/s12913-016-1383-9. PubMed PMID: 27117326; PubMed Central PMCID: PMCPMC4847244.

7. Masulani-Mwale C, Kauye F, Gladstone M, Mathanga D. Prevalence of psychological distress among parents of children with intellectual disabilities in Malawi. BMC Psychiatry. 2018;18(1):146. doi: 10.1186/s12888-018-1731-x. PubMed PMID: 29793452; PubMed Central PMCID: PMCPMC5968565.

8. Scherer N, Verhey I, Kuper H. Depression and anxiety in parents of children with intellectual and developmental disabilities: A systematic review and meta-analysis. PLOS ONE. 2019;14(7):e0219888. doi: 10.1371/journal.pone.0219888.

9. Dambi JM, Jelsma J, Mlambo T. Caring for a child with Cerebral Palsy: The experience of Zimbabwean mothers. Afr J Disabil. 2015;4(1):168. doi: 10.4102/ajod.v4i1.168. PubMed PMID: 28730030; PubMed Central PMCID: PMCPMC5433478 may have inappropriately influenced them in writing this article.

10. Polack S, Adams M, O'Banion D, Baltussen M, Asante S, Kerac M, et al. Children with cerebral palsy in Ghana: malnutrition, feeding challenges, and caregiver quality of life. Dev Med Child Neurol. 2018;60(9):914-21. doi: 10.1111/dmcn.13797. PubMed PMID: 29736993.

11. Developmental difficulties in early childhood. Prevention, early identification, assessment and intervention in low and middle income countries. A review. Geneva: World Health Organisation, 2012.

12. WHO. Meeting report: nurturing human capital along the life course: investing in early child development, 10-11 January 2013. Geneva, Switzerland: World Health Organisations, 2013.

13. G. S, V. L, R. M. Peer support interventions for parents and carers of children with complex needs. . Cochrane Database of Systematic Reviews 2013;(6). doi: 10.1002/14651858.CD010618.

14. McConachie H, Huq S, Munir S, Ferdous S, Zaman S, Khan NZ. A randomized controlled trial of alternative modes of service provision to young children with cerebral palsy in Bangladesh. J Pediatr. 2000;137(6):769-76. doi: 10.1067/mpd.2000.110135. PubMed PMID: 11113832.

15. Zuurmond M, O'Banion D, Gladstone M, Carsamar S, Kerac M, Baltussen M, et al. Evaluating the impact of a community-based parent training programme for children with cerebral palsy in Ghana. PLoS One. 2018;13(9):e0202096. doi: 10.1371/journal.pone.0202096. PubMed PMID: 30180171; PubMed Central PMCID: PMCPMC6122808.

16. Emergency preparedness response. Microcephaly – Brazil. Disease Outbreak News. 8th January 2016. [cited 2019 25/04]. Available from: https://www.who.int/csr/don/8-january-2016-brazil-microcephaly/en/.

17. Mayor S. One in seven children born to mothers who had Zika infection has health problems. BMJ. 2018;362:k3477. doi: 10.1136/bmj.k3477. PubMed PMID: 30101745.
18. de Araujo TVB, Ximenes RAA, Miranda-Filho DB, Souza WV, Montarroyos UR, de Melo APL, et al. Association between microcephaly, Zika virus infection, and other risk factors in Brazil: final report of a case-control study. The Lancet Infectious diseases. 2018;18(3):328-36. Epub 2017/12/16. doi: 10.1016/s1473-3099(17)30727-2. PubMed PMID: 29242091.
19. Leal MC, Muniz LF, Ferreira TS, Santos CM, Almeida LC, Van Der Linden V, et al. Hearing Loss in Infants with Microcephaly and Evidence of Congenital Zika Virus Infection - Brazil, November 2015-May 2016. MMWR Morb Mortal Wkly Rep. 2016;65(34):917-9. doi: 10.15585/mmwr.mm6534e3. PubMed PMID: 27585248.
20. Costello A, Dua T, Duran P, Gulmezoglu M, Oladapo OT, Perea W, et al. Defining the syndrome associated with congenital Zika virus infection. Bull World Health Organ. 2016;94(6):406-A. Epub 2016/06/09. doi: 10.2471/BLT.16.176990. PubMed PMID: 27274588; PubMed Central PMCID: PMCPMC4890216.
21. França GVA, Schuler-Faccini L, Oliveira WK, Henriques CMP, Carmo EH, Pedi VD, et al. Congenital Zika virus syndrome in Brazil: a case series of the first 1501 livebirths with complete investigation. The Lancet. 2016;388(10047):891-7. doi: https://doi.org/10.1016/S0140-6736(16)30902-3.
22. Lowe R, Barcellos C, Brasil P, Cruz OG, Honório NA, Kuper H, et al. The Zika Virus Epidemic in Brazil: From Discovery to Future Implications. International journal of environmental research and public health. 2018;15(1):96. doi: 10.3390/ijerph15010096. PubMed PMID: 29315224.
23. Wheeler AC. Development of Infants With Congenital Zika Syndrome: What Do We Know and What Can We Expect? Pediatrics. 2018;141(Suppl 2):S154-s60. Epub 2018/02/14. doi: 10.1542/peds.2017-2038D. PubMed PMID: 29437048; PubMed Central PMCID: PMCPMC5795516 conflicts of interest to disclose.
24. Kuper H, Smythe T, Duttine A. Reflections on Health Promotion and Disability in Low and Middle-Income Countries: Case Study of Parent-Support Programmes for Children with Congenital Zika Syndrome. International Journal of Environmental Research and Public Health. 2018;15(3). doi: 10.3390/ijerph15030514.
25. Duttine A, Smythe T, Calheiro de Sá M, Ferrite S, Moreira M, Kuper H. Development and assessment of the feasibility of a Zika family support programme: a study protocol [version 1; peer review: 2 approved]. Wellcome Open Research. 2019;4(80). doi: 10.12688/wellcomeopenres.15085.1.
26. Zuurmond M, Nyante G, Baltussen M, Seeley J, Abanga J, Shakespeare T, et al. A support programme for caregivers of children with disabilities in Ghana: Understanding the impact on the wellbeing of caregivers. Child Care Health Dev. 2019;45(1):45-53. doi: 10.1111/cch.12618. PubMed PMID: 30259548.
27. Bowen DJ, Kreuter M, Spring B, Cofta-Woerpel L, Linnan L, Weiner D, et al. How we design feasibility studies. Am J Prev Med. 2009;36(5):452-7. doi: 10.1016/j.amepre.2009.02.002. PubMed PMID: 19362699.
28. Tong A, Sainsbury P, Craig J. Consolidated criteria for reporting qualitative research (COREQ): a 32-item checklist for interviews and focus groups. International Journal for Quality in Health Care. 2007;19(6):349-57. doi: 10.1093/intqhc/mzm042 %J International Journal for Quality in Health Care.
29. Shilling V, Morris C, Thompson-Coon J, Ukoumunne O, Rogers M, Logan S. Peer support for parents of children with chronic disabling conditions: a systematic review of quantitative and qualitative studies. Developmental Medicine & Child Neurology. 2013;55(7):602-9. doi: 10.1111/dmcn.12091.
30. Prost A, Colbourn T, Seward N, Azad K, Coomarasamy A, Copas A, et al. Women's groups practising participatory learning and action to improve maternal and newborn health in low-resource settings: a systematic review and meta-analysis. The Lancet. 2013;381(9879):1736-46. doi: https://doi.org/10.1016/S0140-6736(13)60685-6.

31. Rosato M, Laverack G, Grabman LH, Tripathy P, Nair N, Mwansambo C, et al. Community participation: lessons for maternal, newborn, and child health. The Lancet. 2008;372(9642):962-71. doi: 10.1016/S0140-6736(08)61406-3.
32. Freire P. Pedagogy of the Oppressed. New York: Continuum; 2007.
33. Gram L, Morrison J, Saville N, Yadav SS, Shrestha B, Manandhar D, et al. Do Participatory Learning and Action Women's Groups Alone or Combined with Cash or Food Transfers Expand Women's Agency in Rural Nepal? The Journal of Development Studies. 2018:1-17. doi: 10.1080/00220388.2018.1448069.
