# Supplementary material for: Mothers as facilitators for a parent group intervention for children with Congenital Zika Syndrome: Qualitative findings from a feasibility study in Brazil
Source: PLoS One. 2020 Sep 10;15(9):e0238850. doi: 10.1371/journal.pone.0238850 (PMC7482954; doi:10.1371/journal.pone.0238850)
Supplement: Attachment — Submitted filename: PONE_ReviewerResponse.docx [file pone.0238850.s004.docx]

In this study, the authors use qualitative research methodology to assess the feasibility of a community based intervention that includes using “expert mothers”, whose children also have congenital zika syndrome (CZS), as part of a team to educate families of children with CZS through a series of educational modules. The authors report that the intervention was feasible and that caregivers and expert mothers had favorable opinions on this intervention. This line of research is important because children with CZS may have severe cognitive and motor delays and developing strategies to educate caregivers is essential.

This article benefits from the qualitative research approach and the in-depth responses that were gained from participants. However, the authors could further strengthen this manuscript by a more in-depth discussion of the patient population, the generalizability of this approach in other settings where congenital zika virus exposure occurred, and limitations of the current intervention. My specific revision suggestions are outlined below:

1. The number of parents/caregivers utilized in the study and the actual number of completed questionnaires/interview is somewhat ambiguous. The results section states that 27 families were enrolled, which yielded 38 participants who complete at least one session. They then state that 30 focus groups, 9 participant interviews, and 4 facilitator interviews were conducted. This section would benefit from a more in-depth clarification of the patient population and retention rates:
a) Although 38 participants completed at least one session, how many participants (and families) actually completed the full 10 module sessions? What was the average number of sessions attended by families?
b) What were the attendance rates for different modules and were there better retention for earlier vs later modules. i.e. was there any bias in terms of what information participants were exposed to and could this have affected their responses?
c) How many of the 38 participants completed focus groups and interviews? Results state that there were 30 focus groups, 9 participant interviews, and 4 facilitator interviews. This does not match the numbers listed in “Study Design” section on page 8 were they report 26 focus groups and 13 interviews. Clearly stating the number of responders (and families) would be helpful in understanding the scope of the data.
d) Were participant interviews done individually, so were only 9 out of the 38 participants actually interviewed?
2. The severity of a child’s speech, fine motor, and gross motor disabilities will affect the usefulness of specific training modules. For example, a module on “managing seizures” may not be useful for a family who’s child does not have seizures. Similarly a module on “feeding challenges” will be very different for a child who is fed through a gastrostomy tube vs a child who is fed orally. Additionally the educational and socio-economic status of families will have a tremendous impact on your results. As such, it would be helpful to better understand your patient population in terms of basic patient characteristics:
   a) What proportion of children had mild vs moderate vs severe delay?
   b) What was the prevalence of epilepsy, feeding difficulties, microcephaly, etc. in this patient cohort?
   c) The study states that participants were literate but what was the socioeconomic status of participants and their home/neighborhood environment? Was it mostly patients living in poverty or more middle-class families?

I fully understand if some of this information is not available but having some sense of the patient population would help others understand what groups of patients would most benefit from the proposed approach.

3. Further information on the “expert mother” role would be helpful. Specifically (although the results and discussion sections touch on these topics), the methods section should specify how they were identified, what sort of training they received prior to the teaching sessions, and how often they were from the same neighborhood as the participants.
4. The results section does not describe any concerns or criticisms families or expert mothers may have had about the intervention. The article would thus benefit a breakdown of any negative reactions/limitations categorized by the five elements of feasibility that the authors utilize in their analysis. If there were no negative reactions, there should be a discussion of why that is the case and if there is a positive bias among the responders.
5. The discussion section would benefit from an explicit discussion on the “value added” from expert mothers as it pertains to children with CZS. Are there prior zika-related community interventions that were done without expert mothers? If so, what benefits do you think the addition of an expert mother brings.
6. The discussion section would benefit from a discussion of the challenges encountered in implementing the intervention and approaches the authors took to address these challenges. This would be beneficial for others attempting to implement such interventions.
7. Although the discussion cites other studies where similar approaches have improved patient outcomes, whether the current intervention had long term benefits and improves survival, hospitalization rates, and other outcomes is unclear. Additionally, short term interventions can often suffer from lack of long-term benefits due to their limited nature. The discussion would benefit from expanding the “limitations” paragraph to address these issues and potentially offer suggestions on how the authors could alleviate these limitations moving forward.
