# Supplementary material for: Mothers as facilitators for a parent group intervention for children with Congenital Zika Syndrome: Qualitative findings from a feasibility study in Brazil
Source: PLoS One. 2020 Sep 10;15(9):e0238850. doi: 10.1371/journal.pone.0238850 (PMC7482954; doi:10.1371/journal.pone.0238850)
Supplement: Attachment — Submitted filename: PONE_ReviewerResponse_TS comments.docx [file pone.0238850.s005.docx]

PONE-D-20-02401

Mothers as facilitators for a parent group intervention for children with congenital zika syndrome: qualitative findings from a feasibility study in Brazil

**We thank the reviewers and the Academic Editor for their comments and suggestions and for assisting in the improvement of our manuscript. Our responses are bolded below, with changes to the manuscript underlined. The line numbers refer to the lines on the track changes version.**

Response to Academic Editor:

**We have ensured that we have discussed all potential limitations of this study within the Discussion section, including potential biases introduced by using self-reported data and providing an additional paragraph on implications and future steps.**

**Line 531 – 547 This study has limitations. We explored feasibility of use of two expert mothers in only one setting. We did not compare different strategies of delivery. Nor did we explore in detail what aspects of expert mother were critical to success. Consequently, it is difficult to identify the extent to which the perceived feasibility depended on specific personal characteristics of individual expert mothers, and how much on the use of an expert mother of any kind. The therapist and expert mothers were paid and equal amount and this may have influenced their strong commitment to the programme. Only a subset of participants was selected to participate in semi-structured interviews and this selection, although purposive to gather a range of perspectives, may have introduced a positive reporting bias in the responders. Attendance rates may have influenced participant responses although there was no evidence of better retention for earlier compared to later modules. Furthermore, the selection of interview participants, although done to gather a range of perspectives, may have brought an inherent bias to the qualitative data that was analysed from the participants. However, the triangulation of findings between participants, expert mothers and facilitator therapists gives us confidence in our results. In addition, with a small sample size it is not possible to draw any firm conclusions with regards limited efficacy of the programme or the impact on families of children with different severities of functional impairment.**

**Line 548 Implications and future steps**

**We have identified ongoing research questions that have been framed by the gaps in evidence of this feasibility study. Future studies may seek to establish the cost-effectiveness and long- term benefits (such as improved survival and hospitalization rates) of the inclusion of expert mothers in the delivery of the Juntos intervention. Future research should also investigate the impact of the personal characteristics and experience of the person offering support, and the impact of peer support on caregivers' relationships with health care professionals. In addition, the role of equal payment between the two facilitators warrants further attention.**

**We have included additional information regarding the interview guides used in the manuscript and we provide the topic guides as Supporting Information (S1 and S2). In addition we have created a DOI on the LSHTM repository with the topic guides and will provide this information to the PLOS ONE editorial team should our article be accepted. The study ethics form and consent form specify how we will protect participants and we can to contact the LSHTM ethics/research governance office for a formal response should this be helpful.**

**Reviewer #1:** The topic of the article is relevant and contributes to the literature on Zika. However, the authors need to review detailed edits and comments suggested in the file attached. The methods, results, discussion and conclusion sections need to change significantly because there is confusion on what should be part of each section.

There are several definitions of categories that are included in the results section that should be moved to methods. The results section should incorporate a more in-depth analysis of the findings to substantiate the evidence provided in the practicality, adaption and implementation categories. The acceptability category is much better supported by the evidence provided than the other four categories. I assume that there are more data (narratives from lay experts and participants) to support the analyses of those.

Also, the discussion section should not include results but instead discuss them according to the literature on the subject, which is partially done. There may be other relevant studies that could enrich the discussion of the results.

**Thank you for your review and your suggested changes. We have incorporated these suggestions as follows, and we have:**

1. **clarified sentences and meanings:**

**Line 203 - 204 The interviews were audio recorded by digital sound recorder.**

**Line 202 - 293 The benefits of including the expert mother and reducing the traditional medical hierarchy were also articulated by the therapist facilitator**

**Line 318 - 319 The expert mothers provided an example of what may be possible through their lived experience, which indicates the way the intervention was delivered was appropriate and accessible to caregivers.**

**Line 340 - 341 For example, the expert mothers led the emotional support activity as part of every session, but they did not have a mental health training.**

**Line 467 - 471 A systematic review and meta-analysis of randomised controlled trials undertaken in Bangladesh, India, Malawi, and Nepal of women’s groups practicing participatory learning and action show that these practices improve maternal and neonatal survival [30], by increasing appropriate care-seeking, home prevention and care practices for mothers and new-borns [31].**

**Line 537 – 545 Only a subset of participants was selected to participate in semi-structured interviews and this selection, although purposive to gather a range of perspectives, may have introduced a positive reporting bias in the responders. Attendance rates may have influenced participant responses although there was no evidence of better retention for earlier compared to later modules. Furthermore, the selection of interview participants, although done to gather a range of perspectives, may have brought an inherent bias to the qualitative data that was analysed from the participants. However, the triangulation of findings between participants, expert mothers and facilitator therapists gives us confidence in our results.**

2. **moved the definitions from the results section to methods, specifically**

**Line 219 - 228 Acceptability considers how the recipients react to the inclusion of expert mothers. Demand for the intervention is indicated by the documenting of activities by the expert mother in the caregiver groups. Implementation concerns the extent, likelihood, and manner in which facilitation by the expert mother can be fully implemented as planned and proposed, often in an uncontrolled design. Practicality considers the extent to which facilitation by the expert mother can be implemented when resources, time, commitment, or some combination thereof are constrained in some way. Adaption focusses on how the role of the expert mother may need to be adapted for a new situation. The remaining three areas outlined in the Bowen model of limited efficacy, expansion and integration are to be explored in future studies.**

3. **moved information about participants, training and some information about payment from the results to the materials and methods section where we describe the participants and setting, specifically**

**Line 159 - 166 The facilitators were identified and selected by the study site co-ordinator. Selection characteristics included (i) a similar socioeconomic level to participants, (ii) having a child with a pattern of severity of CZS, (iii) a willingness to help other families, (iv) an active participant in disability rights, and (v) tolerance to personal differences and perspectives (e.g. religion). Both expert mothers were purposively selected by the site co-ordinator, as they had a similar socio-economic level as the group participants, were literate and had completed high school at a minimum as they were required to follow a facilitator manual to deliver the intervention.**

**Line 172 - 177 The role of the therapist was to facilitate the technical aspects of skill acquisition and practice of techniques, such as feeding positions. The therapists and expert mothers completed a joint standardised 5-day facilitators training programme to prepare them for the delivery of Juntos. The expert mother was paid the same as the facilitator therapist (2,100BRL/month, approximately USD500). Transport costs were reimbursed all facilitators and all participants.**

**We have included our findings with regards payment and reimbursement in funds under the results of practicality as these relate to our results:**

**Line 386 – 394 With regards finance, it appeared that equal payment enabled the expert mothers to better facilitate the groups, being more confident of the value of their role in a pair with the therapist. However, the decision to pay expert mothers and therapist facilitators the same amount was initially viewed as unfavourable by the administration team due to precise costing guidelines provided for therapists and relevant experience, with the suggested payment for expert mothers as 1,500BRL/month, approximately USD360. The estimate of salary cost for one therapist to deliver 10 group sessions over three months is 6,300BRL (approximately USD1,500) and the incremental cost of including an expert mother would be 6,300BRL (approximately USD1,500) per programme.**

4. **We include further analysis of the findings pertaining to implementation, practicality and adaption categories and have included these analyses:**

**Implementation:**

**Line 340 – 355 The expert mothers filled their planned role (described above) and this differed from the role of the therapist, who facilitated the technical aspects of skill acquisition, such as feeding positions. However, expert mothers described a tension in their role as a facilitator:**

***“There are some moments where I freeze. It is more difficult that I thought it would be, to be a mother and a facilitator, because I keep pushing myself to act like a therapist during the theoretical moments.”* (Expert mother 01)**

**Both facilitators contributed to practice of techniques. The relationship between the facilitator therapist and the expert mother developed over time through the delivery of the ten session programme and this relationship was sustained by enthusiastic therapists who advocated for family centred services.**

**Interviewer: “*What would you like to do differently in the next session?”***

***“We would like to have a meeting, before the session, to prepare our work together. This will help us to feel more confident to lead the next session.*” (Facilitator therapist 01)**

**Practicality:**

**Line 364 - 385 The practicality of the intervention was facilitated by holding the group sessions in the community, providing transport costs to the expert mothers, paying expert mothers and therapist facilitators equally and supporting the mothers through a mentorship programme. However, certain challenges were identified in the practicality of including mothers as facilitators. There were two occasions when an expert mother was unable to attend. The weekly sessions were held on different week days and the other expert mother was able to assist with one session. In the other session, it was run only by the facilitator therapist. The expert mother in these cases was unable to attend due to her child being ill. Over the three-month period, the majority of caregivers missed at least one session due to illness of their child. Fifteen (60%) families attended seven or more modules. Four families participated in all sessions. Module nine, titled ‘Our Community’, which includes a community day and celebration, was the best attended (group one n = 9; group two n = 7; group three n = 6). The ‘Play and Early Stimulation’ module delivered in week six was the least attended (group one n= 4, group two n = 4, group three n = 3).**

**The group sessions were designed to be held over two to three hours, and typically took approximately four hours. Observation notes suggest that the logistics of delivering the intervention could be challenging, with sessions often starting behind schedule and all the content was not covered in some sessions. As the group facilitators became more familiar with the content, the delivery of the intervention did not exceed the allocated time.**

**Line 395 – 400 The success of the facilitator partnership is likely dependent on both personality and perspective of the pair. Selection characteristics of expert mothers included a similar socioeconomic level to participants and having a child with a severe pattern CZS, with personal characteristics of consideration and understanding. These characteristics may link to a sense of belonging and creation of common ground between the participants.**

**Adaption:**

**Line 402 - 407 Few adaptations were considered to be needed to the role of the expert mother, following the feasibility study, excepting the increased mentoring on providing mental health support, already described. Ongoing weekly mentoring guidelines were developed by the research team, and in partnership with the expert mothers and facilitator therapists, after the first pilot. These guidelines were used to assist the research assistants (psychologists) to provide targeted mentoring for the facilitators to support emotional wellbeing of participants.**

**Line 413 – 436 Resources and processes were refined through the piloting of the ‘Juntos’ intervention and sections of the paper based manual were transformed to video format. These videos were reported as helpful to expert mothers to further demonstrate techniques to participants. Additional resources were requested by the participants and these included:**

***‘We could bring pictures of our shower chairs, or maybe you could show us pictures with the different phases of communication and development of eating, so that we can identify where our children are now, and what the next steps will be – just like we did for the development of moving.”* (Participant 08)**

**There was a need for the study co-ordinator to provide support to facilitators in organisation of logistics e.g. travel, food and intervention materials. The study co-ordinator also provided ongoing instruction, in addition to the comprehensive intervention manual, on how to run practical sessions in order to maximise their impact. Therefore, more practical support may be needed in organizing the sessions in future programmes.**

**When considering future scale up in Brazil, it is likely that adaption of the training to include a greater number of expert mothers compared to the number of facilitator therapists may be required to account for contexts with fewer number of therapists and as mothers may not have the capacity to travel as much or facilitate as many groups as the therapists. The ‘Juntos’ intervention performed in a similar way with all three groups, which consisted of a different population in each group. The co-ordinator selected different sites for the groups and it was reported as important for the expert mother to be local to the community in which the community-based group intervention is run, whilst it is acceptable for the facilitator therapist to travel further and be involved in a greater number of groups.**

Once changes are incorporated in this article, the abstract has to reflect those appropriately. The sentence "One expert mother was unable to attend two sessions due to her child being ill" is a small detail that should not be part of the abstract, for example.

**We have edited our abstract to reflect these changes and we have removed the sentence "One expert mother was unable to attend two sessions due to her child being ill” (Line 53-54).**

**Lines 45 – 56 The use of expert mothers as facilitators was considered to be acceptable and there was demand for their role. Their experiential knowledge was viewed as important for sharing and learning, and supporting and encouraging the group. The intervention was delivered with fidelity by the expert mothers. The practicality of the intervention was facilitated by holding the group sessions in the community, providing transport costs to facilitators and participants, paying expert mothers and therapist facilitators equally and supporting the expert mothers through a mentorship programme. Equal payment with the therapist enabled the expert mothers to better facilitate the groups, through increased confidence in the value of their role. Adaption of the intervention included development of video resources and mentoring guidelines.**

In addition, you need to be more specific in your conclusions, which are too vague. You need to clarify and be more specific about how the use of lay experts could be implemented in Brazil and other low and middle-income countries to address the developmental disabilities of CZS.

**Thank you for your recommendation. We have strengthened our conclusions to clarify how the use of lay experts could be implemented in Brazil, and the generalizability and limitations of this approach.**

**Line 538 – 546 Caregivers with similar life experiences may provide innovate community support to families of children with CZS in resource limited settings. The use of expert mothers in a participatory group setting offers a unique approach to harness the capacity of families to provide care for their child and may be feasible in similar settings. Future consideration for scale up in Brazil includes accounting for resource-limited contexts with fewer number of therapists. It is likely that adaption of the training to include a greater number of expert mothers compared to the number of facilitator therapists may be required.**

**Reviewer 2:**

In this study, the authors use qualitative research methodology to assess the feasibility of a community based intervention that includes using “expert mothers”, whose children also have congenital zika syndrome (CZS), as part of a team to educate families of children with CZS through a series of educational modules. The authors report that the intervention was feasible and that caregivers and expert mothers had favorable opinions on this intervention. This line of research is important because children with CZS may have severe cognitive and motor delays and developing strategies to educate caregivers is essential.

This article benefits from the qualitative research approach and the in-depth responses that were gained from participants. However, the authors could further strengthen this manuscript by a more in-depth discussion of the patient population, the generalizability of this approach in other settings where congenital zika virus exposure occurred, and limitations of the current intervention. My specific revision suggestions are outlined below:

1. The number of parents/caregivers utilized in the study and the actual number of completed questionnaires/interview is somewhat ambiguous. The results section states that 27 families were enrolled, which yielded 38 participants who complete at least one session. They then state that 30 focus groups, 9 participant interviews, and 4 facilitator interviews were conducted. This section would benefit from a more in-depth clarification of the patient population and retention rates:
a) Although 38 participants completed at least one session, how many participants (and families) actually completed the full 10 module sessions? What was the average number of sessions attended by families?

**Thank you for your review of our manuscript and for your suggestions and recommendations. Please find below our point by point responses.**

a. **Thank you for your request for clarification of numbers. 15 families attended more than 7 times. 4 families came to all sessions. Week 9, which included a community day and celebration, was the best attended and Week 6 was the least attended.**

**We have included these data as follows:**

**Line 374 - 379 Over the three-month period, the majority of caregivers missed at least one session due to illness of their child. Fifteen (60%) families attended seven or more modules. Four families participated in all sessions. Module nine, titled ‘Our Community’, which includes a community day and celebration, was the best attended (group one n = 9; group two n = 7; group three n = 6). The ‘Play and Early Stimulation’ module delivered in week six was the least attended (group one n= 4, group two n = 4, group three n = 3).**

b) What were the attendance rates for different modules and were there better retention for earlier vs later modules. i.e. was there any bias in terms of what information participants were exposed to and could this have affected their responses?

**b. We believe that the environment (eg rain or reliable transport) and health of a child both play a role in attendance rate, as well as personal preferences. However, our data are not clear as to the weight that each of these categories contribute to retention.**

**We have therefore included the following comment on bias in the limitations section:**

**Line 512 - 517 We did not compare different strategies of delivery. Nor did we explore in detail what aspects of expert mother were critical to success. Consequently, it is difficult to identify the extent to which the perceived feasibility depended on specific personal characteristics of individual expert mothers, and how much on the use of an expert mother of any kind. The therapist and expert mothers were paid and equal amount and this may have influenced their strong commitment to the programme.**

c) How many of the 38 participants completed focus groups and interviews? Results state that there were 30 focus groups, 9 participant interviews, and 4 facilitator interviews. This does not match the numbers listed in “Study Design” section on page 8 were they report 26 focus groups and 13 interviews. Clearly stating the number of responders (and families) would be helpful in understanding the scope of the data.
d) Were participant interviews done individually, so were only 9 out of the 38 participants actually interviewed?

**c and d. Thank you for your suggestion to clarify numbers, particularly in regard to patient population and retention rates.**

**With regard semi-structured interviews, 13 were undertaken in total, of which 9 were participant interviews (purposively selected from a total of 38 participants – 3 from each group), and 4 were facilitator interviews. In addition, 30 focus group discussions were held, one group after each session. There were 3 groups that ran in total, and each group consisted of 10 sessions. We confirm that 25 children and their families were registered for the intervention and have ensured that this is consistent in both methods and results. We have therefore made the following changes to provide clarification:**

**Line 146 - 149 Data were collected from observation of the caregiver groups (n=30), focus group discussions with caregivers (n=30), and semi-structured interviews with group facilitators (n=4) and purposively selected caregivers (n=9).**

**Line 192 - 199 Third, semi-structured interviews were undertaken by the research assistants with both facilitators of the group and a purposively selected sample of participants, to allow for triangulation of data.**

**Three participants per group were selected after the completion of the ten intervention sessions. Participants were selected at the discretion of the researchers to reflect a broad range of perspectives (e.g. caregivers of children with different severities of disabilities, different caregivers that included mothers, fathers and grandmothers, caregivers of different ages and reflecting a geographic spread).**

2. The severity of a child’s speech, fine motor, and gross motor disabilities will affect the usefulness of specific training modules. For example, a module on “managing seizures” may not be useful for a family who’s child does not have seizures. Similarly a module on “feeding challenges” will be very different for a child who is fed through a gastrostomy tube vs a child who is fed orally. Additionally the educational and socio-economic status of families will have a tremendous impact on your results. As such, it would be helpful to better understand your patient population in terms of basic patient characteristics:
   a) What proportion of children had mild vs moderate vs severe delay?
   b) What was the prevalence of epilepsy, feeding difficulties, microcephaly, etc. in this patient cohort?
   c) The study states that participants were literate but what was the socioeconomic status of participants and their home/neighborhood environment? Was it mostly patients living in poverty or more middle-class families?

I fully understand if some of this information is not available but having some sense of the patient population would help others understand what groups of patients would most benefit from the proposed approach.

**Thank you for your request for additional detail that may assist the reader with generalizability of the approach in other settings. The outline of the modules in Table 1 provides information on the topics provided within the modules. We agree that the severity of a child’s speech, fine motor, and gross motor disabilities may affect the usefulness of specific training modules. However, we believe that the inclusion of peer support activities and psychosocial support within all sessions improves the usefulness of the training modules that may include more specific detail. Whilst there is a module that contains information about ‘managing seizures’, additional information on how to use everyday activities to help your child develop is also included in this module and we believe that the module is therefore useful for all caregivers of children with developmental disabilities. We also agree that the educational and socio-economic status of families will impact the results, and that data on the group population group would assist others in understanding what groups would most benefit from the proposed approach.**

a. **We do not have information on the proportion of the children with mild/moderate or severe delay, however as there was no exclusion criteria based on severity, we include the following information:**

**Line 142 – line 146 Participants were caregivers of children with neurologist-confirmed CZS in Great Salvador, Bahia. For the purposes of this study, we used CZS to describe any child with impairments that can be directly attributed to Zika. The impairments in children included either mild cognitive, communication or functional skill delay and ranged to severe delay in all three developmental categories.**

**b. We do not have the prevalence of epilepsy, feeding difficulties or microcephaly. We have included the following information in our limitations:**

**Line 525 - 527 In addition, with a small sample size it is not possible to draw any firm conclusions with regards limited efficacy of the programme or the impact on families of children with different severities of functional impairment.**

c. **While most patients were from low income households, we did not collect data to determine the socio-economic status of the families for this feasibility study. However, we have included information in the discussion about the need for social support in Brazil**

**Line 496 - 505 A scoping visit by the research team to Salvador, Bahia, prior to piloting the intervention, demonstrated that the majority of support that was provided to families was medically orientated and that informal support networks that were established varied in focus and structure [39]. For example, some groups focussed on advocacy and promoting children’s rights, while many mothers reported being part of WhatsApp groups with other caregivers, which provided some social and emotional support on an ad hoc basis. The addition of an expert mother to the ‘Juntos’ intervention therefore supports the provision of family-centred services through including the sharing of lived experience of caring for a child with CZS. In this study, the role of expert mothers was seen specifically as being important to share and learn together, and to provide support and encouragement.**

3. Further information on the “expert mother” role would be helpful. Specifically (although the results and discussion sections touch on these topics), the methods section should specify how they were identified, what sort of training they received prior to the teaching sessions, and how often they were from the same neighborhood as the participants.

**Thank you for your suggestion. We have included information about participants, training and payment in the materials and methods section where we describe the participants and setting, specifically**

**Line 159 – 166 The facilitators were identified and selected by the study site co-ordinator. Selection characteristics included (i) a similar socioeconomic level to participants, (ii) having a child with a pattern of severity of CZS, (iii) a willingness to help other families, (iv) an active participant in disability rights, and (v) tolerance to personal differences and perspectives (e.g. religion). Both expert mothers were purposively selected by the site co-ordinator, as they had a similar socio-economic level as the group participants, were literate and had completed high school at a minimum as they were required to follow a facilitator manual to deliver the intervention.**

**Line 174 – 177 The therapists and expert mothers completed a joint standardised 5-day facilitators training programme to prepare them for the delivery of Juntos. The expert mother was paid the same as the facilitator therapist (2,100BRL/month, approximately USD500). Transport costs were reimbursed all facilitators and all participants.**

4. The results section does not describe any concerns or criticisms families or expert mothers may have had about the intervention. The article would thus benefit a breakdown of any negative reactions/limitations categorized by the five elements of feasibility that the authors utilize in their analysis. If there were no negative reactions, there should be a discussion of why that is the case and if there is a positive bias among the responders.

**Thank you for your request for further information. We have included results of our analyses of challenges in implementation, practicality and adaption, and we include additional information in the limitations section:**

**Implementation:**

**Line 342 – 346 However, expert mothers described a tension in their role as a facilitator:**

***“There are some moments where I freeze. It is more difficult that I thought it would be, to be a mother and a facilitator, because I keep pushing myself to act like a therapist during the theoretical moments.”* (Expert mother 01)**

**Practicality:**

**Line 367 – 375 However, certain challenges were identified in the practicality of including mothers as facilitators. There were two occasions when an expert mother was unable to attend. The weekly sessions were held on different week days and the other expert mother was able to assist with one session. In the other session, it was run only by the facilitator therapist. The expert mother in these cases was unable to attend due to her child being ill. Over the three-month period, the majority of caregivers missed at least one session due to illness of their child.**

**Line 380 – 383 The group sessions were designed to be held over two to three hours, and typically took approximately four hours. Observation notes suggest that the logistics of delivering the intervention could be challenging, with sessions often starting behind schedule and all the content was not covered in some sessions.** **As the group facilitators became more familiar with the content, the delivery of the intervention did not exceed the allocated time.**

**Adaptions:**

**Line 422 – 426 There was a need for the study co-ordinator to provide support to facilitators in organisation of logistics e.g. travel, food and intervention materials. The study co-ordinator also provided ongoing instruction, in addition to the comprehensive intervention manual, on how to run practical sessions in order to maximise their impact. Therefore, more practical support may be needed in organizing the sessions in future programmes.**

**Limitations**

**Line 513 – 521 Consequently, it is difficult to identify the extent to which the perceived feasibility depended on specific personal characteristics of individual expert mothers, and how much on the use of an expert mother of any kind. The therapist and expert mothers were paid and equal amount and this may have influenced their strong commitment to the programme. Only a subset of participants was selected to participate in semi-structured interviews and this selection, although purposive to gather a range of perspectives, may have introduced a positive reporting bias in the responders. Attendance rates may have influenced participant responses although there was no evidence of better retention for earlier compared to later modules.**

5. The discussion section would benefit from an explicit discussion on the “value added” from expert mothers as it pertains to children with CZS. Are there prior zika-related community interventions that were done without expert mothers? If so, what benefits do you think the addition of an expert mother brings.

**Thank you for your suggestion. We have included a paragraph on value added from expert mothers:**

**Line 496 – 505 A scoping visit by the research team to Salvador, Bahia, prior to piloting the intervention, demonstrated that the majority of support that was provided to families was medically orientated and that informal support networks that were established varied in focus and structure [39]. For example, some groups focussed on advocacy and promoting children’s rights, while many mothers reported being part of WhatsApp groups with other caregivers, which provided some social and emotional support on an ad hoc basis. The addition of an expert mother to the ‘Juntos’ intervention therefore supports the provision of family-centred services through including the sharing of lived experience of caring for a child with CZS. In this study, the role of expert mothers was seen specifically as being important to share and learn together, and to provide support and encouragement.**

6. The discussion section would benefit from a discussion of the challenges encountered in implementing the intervention and approaches the authors took to address these challenges. This would be beneficial for others attempting to implement such interventions.

**Thank you for your suggestion. We have included paragraphs in the results section, of challenged encountered and our resulting actions:**

**Line 380 – 384 The group sessions were designed to be held over two to three hours, and typically took approximately four hours. Observation notes suggest that the logistics of delivering the intervention could be challenging, with sessions often starting behind schedule and all the content was not covered in some sessions. As the group facilitators became more familiar with the content, the delivery of the intervention did not exceed the allocated time.**

**Line 442 – 426 There was a need for the study co-ordinator to provide support to facilitators in organisation of logistics e.g. travel, food and intervention materials. The study co-ordinator also provided ongoing instruction, in addition to the comprehensive intervention manual, on how to run practical sessions in order to maximise their impact. Therefore, more practical support may be needed in organizing the sessions in future programmes.**

**We have also included a paragraph of comparison of findings in the discussion:**

**Line 481 – 494 Comparison of our findings is also possible with other interventions for children with developmental disabilities in different low resourced settings , such as: (i) a Caregiver Skills Training (CST) for caregivers of children with intellectual disabilities [34, 35], developed by the World Health Organisation, (ii) ‘Titukulane’, an eight module community group intervention that aims provide contextualised psychological support to caregivers of children with intellectual disabilities [36], (iii) Learning through Everyday Activities with Parents (LEAP-CP), which aims to improve the mobility of children with cerebral palsy over 30 weekly peer-to-peer home visits [37], and (iv) PASS, a parent-mediated intervention for autism spectrum disorder in India and Pakistan [38] that was adapted for delivery by non-specialist workers and uses video feedback methods to address parent–child interaction. The focus on caregiver involvement is a common thread in all of these interventions, which is critical, particularly where there are few health services. These interventions demonstrate that reaching family-centred care goals can be facilitated through having mothers as facilitators. However, formal evaluation of their effectiveness and cost-effectiveness is lacking.**

7. Although the discussion cites other studies where similar approaches have improved patient outcomes, whether the current intervention had long term benefits and improves survival, hospitalization rates, and other outcomes is unclear. Additionally, short term interventions can often suffer from lack of long-term benefits due to their limited nature. The discussion would benefit from expanding the “limitations” paragraph to address these issues and potentially offer suggestions on how the authors could alleviate these limitations moving forward.

**Thank you for your suggestion. We have included ‘implications and future steps’ in our limitations section:**

**Line 529 – 535 We have identified ongoing research questions that have been framed by the gaps in evidence of this feasibility study. Future studies may seek to establish the cost-effectiveness and long- term benefits (such as improved survival and hospitalization rates) of the inclusion of expert mothers in the delivery of the Juntos intervention. Future research should also investigate the impact of the personal characteristics and experience of the person offering support, and the impact of peer support on caregivers' relationships with health care professionals. In addition, the role of equal payment between the two facilitators warrants further attention.**
