# Supplementary material for: Mothers as facilitators for a parent group intervention for children with Congenital Zika Syndrome: Qualitative findings from a feasibility study in Brazil
Source: PLoS One. 2020 Sep 10;15(9):e0238850. doi: 10.1371/journal.pone.0238850 (PMC7482954; doi:10.1371/journal.pone.0238850)
Supplement: Attachment — Submitted filename: PONE_ReviewerResponse_2.docx [file pone.0238850.s006.docx]

PONE-D-20-02401

Mothers as facilitators for a parent group intervention for children with congenital zika syndrome: qualitative findings from a feasibility study in Brazil

**We thank the reviewers and the Academic Editor for their comments and suggestions and for assisting in the improvement of our manuscript. Our responses are bolded below, with changes to the manuscript underlined. The line numbers refer to the lines on the track changes version.**

Response to Academic Editor and Reviewer #1: Small edits are needed to standardize writing of some words, such as newborns, ongoing, etc. Adaption is not a word in English. It should be Adaptation. The article still needs revision of the English to fix a few issues.

**We have ensured that the article is proofread and the spelling and grammar amended. We have corrected the following:**

**Line 43: and adaptation**

**Line 53 – 54: Adaptation of the intervention included development of video resources and mentoring guidelines**

**Line 85 – 86 are recommended by the WHO to reduce newborn mortality and improve health in low resource settings**

**Line 202 – 203: Specifically, we focussed on the facets of acceptability, demand, implementation, practicality and adaptation of the delivery of the**

**Line 211 – 212: Adaptation focusses on how the role of the expert mother may need to be adapted for a new situation.**

**Line 371 – 373 Adaptation. Few adaptations were considered to be needed to the role of the expert mother, following the feasibility study, excepting…**

**Line 374 Weekly mentoring guidelines were developed by the research team, and in partnership with the expert mothers and facilitator therapists, after the first pilot.**

**Line 387 – 388 The study co-ordinator also provided instruction, in addition to the comprehensive intervention manual…**

**Line 391 – 392 When considering future scale up in Brazil, it is likely that adaptation of the training to include a greater number of expert mothers compared to the number of facilitator therapists**

**Line 471 – 472 We have identified research questions that have been framed by the gaps in evidence of this feasibility study.**

**Line 483 – 485 It is likely that adaptation of the training to include a greater number of expert mothers compared to the number of facilitator therapists may be required.**
